# Leveraging Clinical Digitized Data to Understand Temporal Characteristics and Outcomes of Acute Myocardial Infarctions at a Tertiary Care Medical Centre in Pakistan from 1988–2018 – Methods and Results

**DOI:** 10.5334/gh.1147

**Published:** 2022-08-18

**Authors:** Zainab Samad, Ali Aahil Noorali, Awais Farhad, Safia Awan, Nada Qaiser Qureshi, Minaz Mawani, Mushyada Ali, Laiba Masood, Ghufran Adnan, Linda K. Shaw, Fahim Haider Jafary, Salim S. Virani, Eric J. Velazquez, Zulfiqar Bhutta, Gerald S. Bloomfield, Javed Tai

**Affiliations:** 1 Department of Medicine, Aga Khan University, Karachi, Pakistan; 2 Division of Cardiology, Department of Medicine, Duke University, Duke Global Health Institute, Duke Clinical Research Institute, Durham, NC, USA; 3 Department of Epidemiology and Biostatistics, College of Public Health, University of Georgia, Athens, Georgia, USA; 4 Independent Biostatistical Consultant, USA; 5 Department of Cardiology, Tan Tock Seng Hospital, Singapore; 6 Division of Cardiology, Department of Medicine, Baylor College of Medicine, US; 7 Michael E. DeBakey Veterans Affairs Medical Centre, Houston TX, USA; 8 Section of Cardiology, Department of Medicine, Yale School of Medicine, Yale University, New Haven, CO, USA; 9 Institute of Global Health and Development, Aga Khan University, Karachi, Pakistan

**Keywords:** Myocardial Infarction, Electronic Health Records, Health Care Outcome Assessment, Quality of Health Care, Risk Factors

## Abstract

**Background and Objective:**

Few data exist on trends in acute myocardial infarction (AMI) patterns spanning recent epidemiological shifts in low middle-income countries (LMICs). To understand temporal disease patterns of AMI characteristics and outcomes between 1988–2018, we used digitized legacy clinical data at a large tertiary care centre in Pakistan.

**Methods:**

We reviewed digital health information capture systems maintained across the Aga Khan University Hospital and obtained structured elements to create a master dataset. We included index admissions of patients >18 years that were discharged between January 1, 1988, and December 31, 2018, with a primary discharge diagnosis of AMI (using ICD-9 diagnoses). The outcome evaluated was in-hospital mortality.

Clinical characteristics derived from the electronic database were validated against chart review in a random sample of cases (*k* 0.53–1.00).

**Results:**

The final population consisted of 14,601 patients of which 30.6% (n = 4,470) were female, 52.4% (n = 7,651) had ST elevation MI and 47.6% (n = 6,950) had non-ST elevation MI. The median (IQR) age at presentation was 61 (52–70) years. Overall unadjusted in-hospital mortality was 10.3%. Across the time period, increasing trends were noted for the following characteristics: age, proportion of women, prevalence of hypertension, diabetes, proportion with NSTEMI (all p_trend_ < 0.001). In-hospital mortality rates declined significantly between 1988–1997 and 2008–2018 (13.8% to 9.2%, p < 0.001).

**Conclusions:**

The patterns of AMI have changed over the last three decades with a concomitant decline in in-hospital mortality at a tertiary care centre in Pakistan. Clinical digitized data presents a unique opportunity for gaining insights into disease patterns in LMICs.

## Introduction

The burden of ischemic heart disease is rising disproportionately in low middle income countries (LMICs) such as Pakistan [1]. Acute myocardial infarction (AMI) is the archetype cardiovascular emergency [2]. Outcomes of AMI have improved substantially in developed countries and are often rigorously tracked through national registries and health system databases to serve as guides for continuous improvement efforts [3,4]. However, while the need is great in LMICs, such registries and tracking efforts are scarce [5,6]. In addition there are few research studies reporting on outcomes of AMI from LMICs.

Whereas electronic health record (EHR) powered databases have been used extensively to understand disease outcomes trends in developing countries, few examples exist in LMICs due to resource constraints, inadequacy of digitized clinical data and lack of linkages among public-sector maintained resources [7]. The utilization of such digitized clinical data, acquired as a by-product of clinical care across large time periods and across various data capture systems, in LMICs, for research and quality improvement purposes has not been explored at scale previously.

We aimed to leverage longitudinally maintained hospital clinical data at a tertiary care centre in Pakistan to address knowledge gaps around trends in AMI patterns over the last three decades. We hypothesized that digitized data could be leveraged to understand AMI characteristics and outcomes, and that AMI mortality had declined over time [8].

## Methods

**Study Setting:** Our study was carried out at the Aga Khan University Hospital in Karachi, Pakistan.

**Patient Population:** We included patients >18 years with a primary discharge diagnosis of acute myocardial infarction between January 1, 1988, to December 31, 2018. Only index admissions with a primary discharge diagnosis of acute myocardial infarction were used for all analyses.

### Data Curation and Analysis

**Figure 1:**
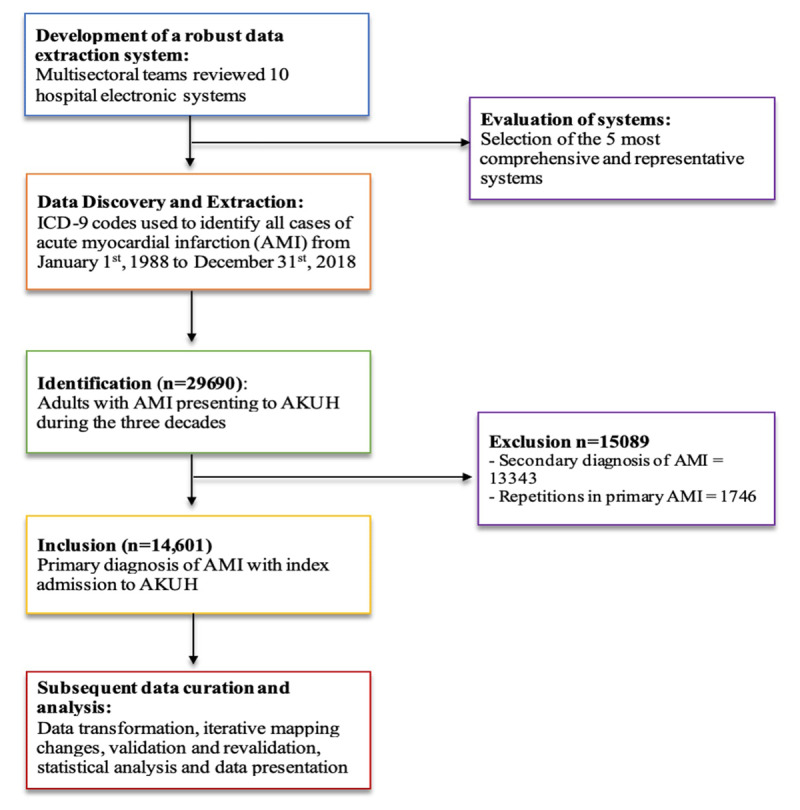
Schematic process flowchart highlighting the data management stratagem and study enrolment chart using STROBE guidelines.

Although inpatient admissions and care is mostly documented using paper charts at AKUH, this is supplemented by a robust health information technology support structure that captures structured data elements that AKUH has maintained since its inception. For this study we explored and leveraged the data sources at the Aga Khan University Hospital (AKUH) in Karachi. Our methodological approach can be divided into three overarching strategic phases: 1) Data discovery and extraction, 2) Data transformation 3) Data validation (Figure 1).

#### (1) Data Discovery and Extraction

The Aga Khan University Hospital Information Management System (HIMS) has been prospectively maintaining data at the Aga Khan University Hospital since 1988. This department populates data on each patient after a rigorous review of the chart by master coders after patient discharge at the conclusion of a hospital admission. As part of its routine practice the HIMS departments re-evaluates 15% of daily discharges (selected randomly) for quality assurance of coding accuracy in the Abstracting System with a benchmark target of 97.5%. Multiple digital systems comprise the hospital information system and after an initial review of ten digital data capture systems we used five systems for the extraction process, based on their clinical relevance, and potential for quality improvement intervention.

The Abstracting System contains primary, secondary, and associated diagnoses using International Classification of Diseases, 9th revision (ICD-9) codes and ICD-9 procedure codes and has been maintained since 1988. This system was used to identify the AMI cases. We used the ICD-9 diagnoses codes of 410.0 – 410.9, 410.00 – 410.02, 410.10 – 410.12, 410.20 – 410.22, 410.30 – 410.32, 410.40 – 410.42, 410.50 – 410.52, 410.60 – 410.62, 410.70 – 410.72, 410.80 – 410.82 and 410.90 – 410.92 to identify cases of AMI. NSTEMI was defined as diagnosis codes 410.7 and 410.70 – 410.72 and STEMI was defined as diagnoses codes 410.0 – 410.6, 410.8 – 410.9, 410.00 – 410.02, 410.10 – 410.12, 410.20 – 410.22, 410.30 – 410.32, 410.40 – 410.42, 410.50 – 410.52, 410.60 – 410.62, 410.80 – 410.82 and 410.90 – 410.92. In addition, associated clinical diagnoses reporting on diseases such as diabetes, hypertension, dyslipidaemia, and AMI complications were also curated on the patients with AMI. PCI was defined as ICD-9 procedure codes 00.66, 36.06 and 36.07, CABG was defined as procedure codes 36.10–36.16, and angiography was defined as procedure codes 37.21–37.23 and if PCI was done.

The presence of a diagnosis code reporting the condition on discharge was considered presence of the condition and absence of the diagnosis code as absence of a history of the condition. From the extracted data set we included index admissions where the primary discharge diagnosis was an AMI as defined by the above codes. Repeat admissions and records identifying AMI as a secondary diagnosis and (n = 15,089) were excluded (Figure 1).

#### (2) Data transformation

The transformation process was designed such that raw data from the source files were imported. Mappings were first done for a pilot dataset of one year. Analysis of this data resulted in finding rectifiable errors in the mapping process and subsequent iterative changes in the transformation rules that were then applied to the entire data set spanning 1988–2018.

#### (3) Data validation

A paper chart review of discharge summaries and inpatient notes was conducted by three independent reviewers. These reviewers were blinded to the digital dataset at the time of the chart review. For the purposes of this validation process, a random sample of 1.0% electronic records from the 30-year dataset was identified for the chart review process but paper chart review was possible on 0.34% of the cohort as some paper files, purged based on hospital policies, were not available for review. The age field was extracted from the abstracting system which maintains the most up to date information on demography of any given patient. The age in the system is calculated using date of admission subtracted from the date of birth rounded to year.

### Patient and Public Involvement

Patient and public were not directly involved in the design or execution of this project.

### Statistical analysis

For data validation, Cohen’s kappa coefficient was calculated for categorical variables (chart review vs digital dataset) and ICC was used to describe agreement in continuous variables between chart review and the digital dataset.

Continuous variables such as age and length of hospital stay were described using median with interquartile ranges. Categorical variables were described as proportions with percentages. The cohort was described as a whole, by STEMI and NSTEMI, and by time periods (1988–1997, 1998–2007 and 2008–2018). Differences in continuous variables were examined using Generalized Linear Models and reported the p value of likelihood ratio chi-square test. Likewise, differences in categorical variables were evaluated using the linear-by-linear chi-square statistic. Statistical analyses were carried out using Statistical Package for Social Sciences (SPSS, version 19.0).

This study was approved by the Ethical Review Committee at the Aga Khan University (2018-0149-219).

## Results

### Cohort Identification and Validation

A total of 29,690 discharges with AMI were identified during the period 1988–2018. Of these 14,601 comprised index admissions on unique patients discharged/deceased with a primary discharge diagnosis of AMI (Figure 1). The Cohen’s kappa coefficient for categorical variables such as patient’s demographics, diagnoses (primary and associated) and procedures ranged from 0.53 to 1.00. ICC for length of hospital stay and age was 0.95 and 0.99 respectively (Table 1).

**Table 1:** Validation of categorical variables (30-year dataset); n = 49.


CATEGORICAL VARIABLES	OBSERVED AGREEMENT (%)	ĸ STATISTIC

Sex	97.9%	0.95

AMI type (STEMI vs NSTEMI)	97.9%	0.94

Smoker	91.8%	0.80

Diabetes Mellitus	95.9%	0.91

Hypertension	100.0%	1.00

Dyslipidaemia	97.9%	0.93

Coronary Angiography	95.9%	0.91

Percutaneous Coronary Intervention	97.9%	0.95

Coronary Artery Bypass Graft	100.0%	1.00

Acute Kidney Injury	83.6%	0.53

Cardiogenic Shock	97.9%	0.79

Mortality	100.0%	1.00

**CONTINUOUS VARIABLES**	**INTRA-CLASS CORRELATION**	**95% CI**

Age	0.99	0.98–0.99

Length of hospital stay	0.95	0.91–0.97


### Population Characteristics

Among 14,601 patients with AMI, the median (interquartile range) was 61 (52–70) years, and 10,131 (69.4%) patients were men. A total of 1,315 (9.0%) patients were <45 years of age; n = 7298 (50.0%) were between 45–64 years of age; and 5,988 (41.0%) patients were 65 years of age. Women were older compared with men (64.5 years. vs. 59.3 years respectively, p < 0.001). A total of 7,651 (52.4%) patients had STEMI and 6,950 (47.6%) patients had NSTEMI (Table 2).

**Table 2:** Total and decade-wise chronological trends of patient characteristics (n = 14,601).


	1988–2018 TOTAL n = 14,601 n (%)	1988–1997 TOTAL n = 2544 n (%)	1998–2007 TOTAL n = 5102 n (%)	2008–2018 TOTAL n = 6955 n (%)	*P*_trend_

Age; Median (IQR)	61 (52–70)	58 (50–66)	60 (52–70)	63 (54–71)	<0.001*

Range	19–101	22–94	19–96	19–101	

Sex					

Male	10131 (69.4)	1859 (73.1)	3603 (70.6)	4669 (67.1)	<0.001

Female	4470 (30.6)	685 (26.9)	1499 (29.4)	2286 (32.9)	

STEMI	7651 (52.4)	2076 (81.6)	2714 (53.2)	2861 (41.1)	<0.001

NSTEMI	6950 (47.6)	468 (18.4)	2388 (46.8)	4094 (58.9)	

**RISK FACTORS**					

Current Smoker	4388 (30.1)	336 (13.2)	1842 (36.1)	2210 (31.8)	<0.001

Diabetes Mellitus	6985 (47.8)	962 (37.8)	2342 (45.9)	3681 (52.9)	<0.001

Hypertension	9070 (62.1)	1108 (43.6)	3006 (58.9)	4956 (71.3)	<0.001

Dyslipidaemia	3594 (24.6)	284 (11.2)	2264 (44.4)	1046 (15.0)	<0.001

**PROCEDURES**					

Coronary Angiography	7382 (50.6)	110 (4.3)	2699 (52.9)	4573 (65.8)	<0.001

Percutaneous Coronary Intervention	4626 (31.7)	17 (0.7)	1366 (26.8)	3243 (46.6)	<0.001

Coronary Artery Bypass Graft	1149 (7.9)	16 (0.6)	546 (10.7)	587 (8.4)	<0.001

**COMPLICATIONS AND OUTCOMES**					

Acute Kidney Injury	2295 (15.7)	78 (3.1)	347 (6.8)	1870 (26.9)	<0.001

Cardiogenic Shock	1206 (8.3)	142 (5.6)	373 (7.3)	691 (9.9)	0.001

Heart Failure	4129 (28.3)	684 (26.9)	1422 (27.9)	2023 (29.1)	0.072

In-hospital Mortality	1507 (10.3)	351 (13.8)	513 (10.1)	643 (9.2)	<0.001

Length of hospital stay (days); median (IQR)	4 (3–7)	6 (4–8)	5 (3–8)	3 (2–6)	<0.001*


* p value of likelihood ratio chi-square test.

### Temporal Trends

**Figure 2:**
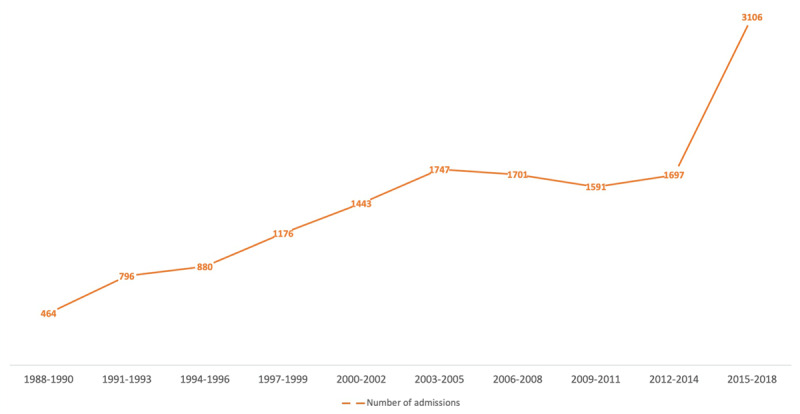
Trends in total number of AMI index admissions.

**Figure 3:**
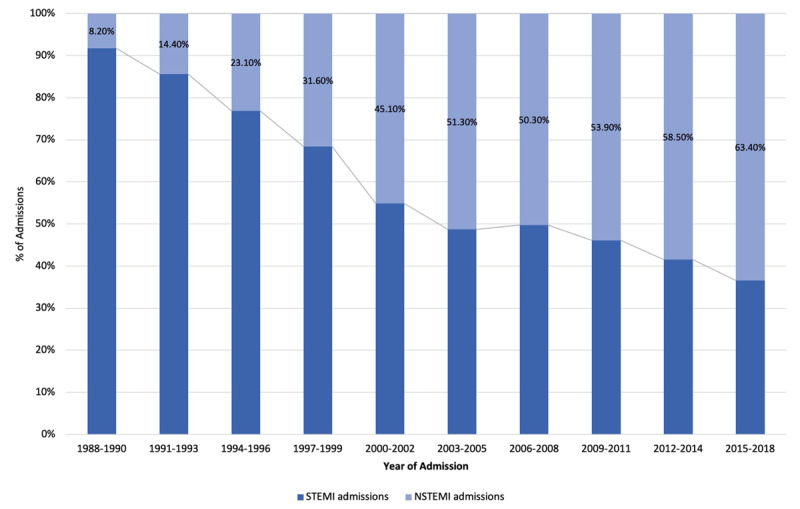
Trends in proportions of STEMI and NSTEMI.

Between 1988–2018, a rise in yearly AMI admissions was noted (Figure 2). In addition, a rising trend was observed among all risk factors such as smoking, diabetes, hypertension, dyslipidaemia (p < 0.001). Over the three decades (1^st^ vs 2^nd^ vs 3^rd^), a significant increase was observed in the proportion of women presenting with AMI (26.9% vs 29.4% vs 32.9%, p < 0.001). The proportion of STEMI declined over the years from 81.6% during first decade to 41.1% during last decade and a rise in the proportion of NSTEMI admissions was concomitantly observed (Table 3 and Figure 3). The median age at presentation of STEMIs increased from 57 years to 59 years. Patients with NSTEMI were older and their median age at presentation increased from 61 years to 65 years (Table 3).

**Table 3:** Temporal trends of characteristics of STEMI (n = 7,651) and NSTEMI (n = 6,950).


STEMI (N = 7,651)				

	1988–1997 TOTAL n = 2076 n (%)	1998–2007 TOTAL n = 2714 n (%)	2008–2018 TOTAL n = 2861 n (%)	*P*_trend_

Age, in years, median (IQR)	57 (49–65)	58 (50–66)	59 (50–68)	<0.001*

Range	22–94	25–96	19–100	

Sex				

Male	1567 (75.5)	2092 (77.1)	2134 (74.6)	0.360

Female	509 (24.5)	622 (22.9)	727 (25.4)	

**RISK FACTORS**				

Current Smoker	241 (11.6)	1086 (40.0)	1031 (36.0)	<0.001

Diabetes Mellitus	759 (36.6)	1090 (40.2)	1250 (43.7)	<0.001

Hypertension	851 (41.0)	1386 (51.1)	1735 (60.6)	<0.001

Dyslipidaemia	201 (9.7)	1192 (43.9)	412 (14.4)	0.383

**PROCEDURES**				

Coronary Angiography	77 (3.7)	1626 (59.9)	2477 (86.6)	<0.001

Percutaneous Coronary Intervention	12 (0.6)	961 (35.4)	2088 (73.0)	<0.001

Coronary Artery Bypass Graft	12 (0.6)	267 (9.8)	223 (7.8)	<0.001

**COMPLICATIONS AND OUTCOMES**				

Acute Kidney Injury	65 (3.1)	148 (5.5)	508 (17.8)	<0.001

Cardiogenic Shock	130 (6.3)	287 (10.6)	462 (16.1)	<0.001

Heart Failure	530 (25.5)	523 (19.3)	495 (17.3)	<0.001

In-hospital Mortality	314 (15.1)	334 (12.3)	331 (11.6)	<0.001

Length of hospital stay (days); median (IQR)	6 (4–8)	4 (3–7)	3 (2–5)	<0.001*

**NSTEMI (N = 6,950)**				

	**1988–1997** **TOTAL n = 468** **n (%)**	**1998–2007** **TOTAL n = 2388** **n (%)**	**2008–2018** **TOTAL n = 4094** **n (%)**	***P*_trend_**

Age; in years, median (IQR)	61 (53–69)	64 (55–72)	65 (57–74)	<0.001*

Range	25–90	20–96	19–101	

Sex				

Male	292 (62.4)	1511 (63.3)	2535 (61.9)	0.418

Female	176 (37.6)	877 (36.7)	1559 (38.1)	

**RISK FACTORS**				

Current Smoker	95 (20.3)	756 (31.7)	1179 (28.8)	0.289

Diabetes Mellitus	203 (43.4)	1252 (52.4)	2431 (59.4)	<0.001

Hypertension	257 (54.9)	1620 (67.8)	3221 (78.7)	<0.001

Dyslipidaemia	83 (17.7)	1072 (44.9)	634 (15.5)	<0.001

**PROCEDURES**				

Coronary Angiography	33 (7.1)	1073 (44.9)	2096 (51.2)	<0.001

Percutaneous Coronary Intervention	5 (1.1)	405 (17.0)	1155 (28.2)	<0.001

Coronary Artery Bypass Graft	4 (0.9)	279 (11.7)	364 (8.9)	0.130

**COMPLICATIONS AND OUTCOMES**				

Acute Kidney Injury	13 (2.8)	199 (8.3)	1362 (33.3)	<0.001

Cardiogenic Shock	12 (2.6)	86 (3.6)	229 (5.6)	<0.001

Heart Failure	154 (32.9)	899 (37.6)	1528 (37.3)	<0.001

In-hospital Mortality	37 (7.9)	179 (7.5)	312 (7.6)	0.973

Length of hospital stay (days); median (IQR)	6 (4–8)	5 (3–8)	3 (2–6)	<0.001*


* p value of likelihood ratio chi-square test.

**Figure 4:**
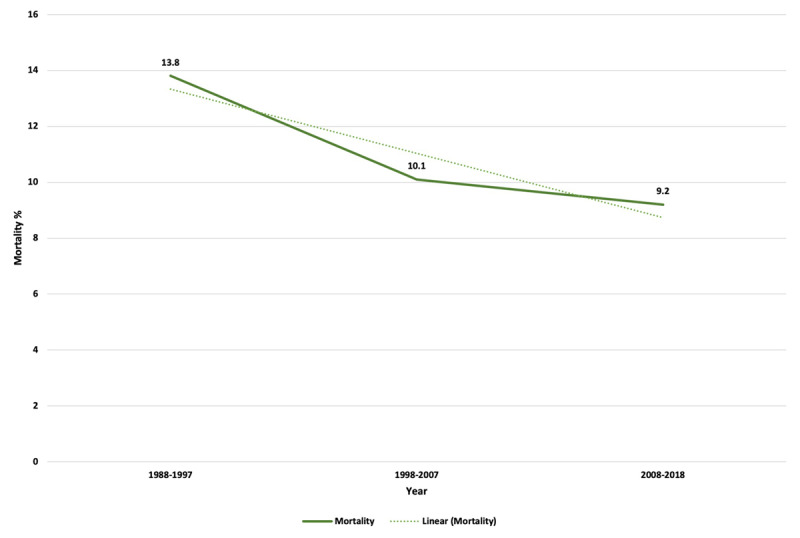
Chronological trends of AMI mortality over three decades.

Overall, the proportion of patients receiving PCI, angiography and CABG rose over the years. Concomitantly from 1988 to 2018, a downward trend in length of stay (median six days in first decade to three days in the last decade) and in-hospital mortality (Figure 4) from 13.8% during first decade to 9.2% in the last decade (*p*_trend_ < 0.001). A significant decline in in-hospital mortality was observed over the three decades (1^st^ vs 2^nd^ vs 3^rd^) for STEMI (15.1% vs 12.3% vs 11.6%, p < 0.001) but not for patients with NSTEMI (7.9% vs 7.5% vs 7.6%, p = 0.97) (Table 3).

## Discussion

This study is the first description of the use of digitized legacy clinical data to understand temporal disease patterns at a large tertiary centre in Pakistan. Digitized data were transformed and successfully validated against chart review, and the main findings include the following: 1) AMI admissions have steadily increased over the last three decades, 2) the age at presentation has steadily increased, 3) the distribution of MIs has changed with increasing NSTEMIs and decreasing STEMIs over time, and 4) In-hospital mortality has decreased with time.

### 1) AMI admissions have steadily increased over the last three decades

According to the 2017 Census by the Pakistan Bureau of Statistics, Pakistan has <1300 hospitals serving a population of 207.78 million individuals [9]. AKUH is one of the foremost tertiary care hospitals in the region accredited by the Joint Commission International and has a catchment population reach of 11 million within a 15km radius [10].

AKUH caters to patients with diverse ethnic and socioeconomic backgrounds and so trends noted in our study may be generalizable to the catchment population. The present study examining trends across three decades demonstrates an increased number of AMI admissions. Similar temporal trends have been highlighted in New Zealand where the hospitalizations for AMI have increased by 8% from 1993 to 2005 [11]. However, most developed countries are experiencing a decline in AMI incidence rates [12]. The increasing trends in admissions in our study possibly reflect on improved diagnostic capabilities with more diagnoses of AMI but also more likely the growing cardiovascular disease burden in Pakistan and other low middle-income countries [13,14]. The increase in CVD burden in LMICs has been attributed to increased burden of smoking and increasing prevalence of risk factors such as hypertension and diabetes that were also observed in our cohort [15].

### 2) Increasing age at presentation, but younger than developed countries

The median age at presentation in our study was 58 (50–66) in the first decade and increased to 63 (54–71) in the last decade. Similar trends were seen across 2,157 US hospitals in the National Registry of Myocardial Infarction (NRMI), where mean age at presentation increased from 64.1 in 1990 to 66.4 in 2006 [16]. In the SWEDEHEART registry reporting on STEMIs from 1995 to 2014, the mean age at presentation was 70 years [17]. Similarly in a study from UK across 243 hospitals in England and Wales the median age at presentation of AMI was 70.0 years [18]. Our STEMI cohort was more than a decade younger than in the US and Europe and even though age increased with time, the median age at presentation for STEMI remained in the 50s over the last decade. This age difference could be attributed to a more aggressive and more premature phenotype of coronary artery disease in South Asia, complex gene-environment interplays and a higher burden of metabolic risk factors and comorbid diagnoses such as prior diabetes, hypertension at younger ages in South Asian patients [19,20,21].

### 3) Temporal changes in STEMI and NSTEMI proportions and risk factor burdens

Concomitant to increasing age at presentation, the proportion of STEMIs declined and NSTEMIs increased over time. The lower proportion of NSTEMIs during the first decade is likely due to non-availability of more specific and highly sensitive biomarkers such as high sensitivity troponin. Similar trends have been noted in other countries such as the NRMI in the United States (n = 1,950,561) where the proportion of NSTEMI showed a significant jump from 14.2% in 1990 to 59.1% in 2006 and the French registry (FAST-MI, n = 14,223) projecting a similar jump from 29% in 1995 to 51% in 2015 [16,22]. The Korea Acute Myocardial Infarction study also showed a decrease in incidence of STEMI from 60.5% in 2006 to 48.1% in 2013, along with an increase in incidence of NSTEMI from 39.5% in 2006 to 59.1% in 2013 [23]. This MI phenotypic shift may be related to risk factor prevalence and background risk factor treatment.

The complexity of patients increased over time with higher risk factor burden and higher prevalence of AMI complications including acute kidney injury and cardiogenic shock [24]. It is possible that the decrease in STEMI over time could be attributable to distribution of such patients across other hospitals in the city with the gradual initiation of primary PCI programs in nine institutions across the city, including free PCI management at public sector hospitals. Despite that, the in-hospital mortality of STEMI is lower during the last decade primarily due to availability of primary PCI for all eligible patients.

The unusually low prevalence of dyslipidemia during the first and the last decade is likely due to changes in clinical practice. During the first decade, lipid panels were not routinely checked. During the second decade, it was standard practice for all patients to have their lipids checked following admission to the hospital. However, during recent years, lipid panels are not routinely checked during hospitalization, but rather checked 12 weeks after initiation of high intensity statin therapy.

Contrastingly, with diabetes, we noted both a high prevalence and an increasing trend across the decades. Data from the International Diabetes Federation’s Atlas (10th Edition) shows that Pakistan ranks third in the number of adults (20–79 years) with diabetes, and first in age-adjusted comparative diabetes prevalence in adults [25]. Further on, data from the National Diabetes Survey of Pakistan (NDSP), 2016–2017, indicates a high overall weighted prevalence of diabetes (26.3%) in the Pakistani population [26]. Given that South Asian countries, particularly Pakistan, have a drastically high prevalence of diabetes compared to other countries, and that our source population is patients with acute myocardial infarction rather than the overall community, the reported diabetes prevalence within this cohort is notably high.

### 4) In-hospital mortality decreased over time, and this was likely attributed to a drop in STEMI related mortality

Across the study period, in-hospital mortality decreased from 15.2% in 1988–1990 to 8.2% in 2016–2018 (p < 0.001). Moreover, there was a significant decline in the in-hospital mortality rates between 1988–1997 and 2008–2018 (13.8% to 9.2%, p < 0.001). Large datasets from Japan (n = 27,220), Italy (1,110,822) and the United States (46,086) have shown a similar decline in AMI mortality over the decades [27,28,29]. A recent cross-sectional study in Pakistan has shown comparable mortality statistics. This two-year study, spanning 11 territory care hospitals in the Punjab province of our country, found the AMI mortality to be 8.6% [30]. Together, these data suggest that although variability exists, there has been an overall decrease in AMI mortality, but there is still room for improvement. In addition to institutional guideline directed therapy, factors such as improving competencies of healthcare workers’ training, awareness of cardiovascular diseases and their risk factors, and growth of the cardiology workforce may have contributed to these reductions.

Overall, AMI in-hospital mortality decline was likely attributable to a drop in STEMI related in-hospital mortality. There was no significant change in NSTEMI mortality. The decline in STEMI mortality is likely related to increased use of revascularization and guideline directed medical therapy over time. During the first decade, patients were primarily treated medically, including patients with STEMI who received lytic therapy. Although the first cardiac catheterization laboratory at AKUH was constructed in 1995, PCI was first performed in 1996, and it was not until 1999 that primary PCIs were performed, and a 24/7 primary PCI program (first in country) was established in 2006. This is the probable reason why only 0.7% patients underwent PCI during the first decade. Even though PCI rates increased over time, they remained <80% in the last decade.

### Impact

This study provides important evidence on the changing patterns of AMI in a low middle-income country. It also highlights the need for tracking AMI outcomes and care through a registry/quality-network approach in other tertiary care hospitals in Pakistan and other LMICs. Through this dataset, clinicians can put into perspective outcome gains over three decades at a tertiary medical centre in Pakistan. These data need to be put in context with other data gathering efforts at larger network of hospitals across Pakistan to contribute to quality improvement through novel interventions in therapy, coordination of systems and health literacy. Any subsequent quality improvement interventions in lifestyle, emergency management and systems’ development must have greater availability, accessibility, awareness, and affordability as core foundations.

Our use of lean digitized clinical data longitudinally maintained represents a novel approach in an LMIC. This study demonstrates the value of clinical data capture that can be accomplished in most medical centres in LMICs. Employing electronic data capture in health facilities in Pakistan and other LMICs has substantial potential implications for benchmarking, strategy development, quality improvement and policy reform in LMICs [31,32,33]. Indeed, it is often the lack of timely reliable data that acts as a serious impediment to health care delivery [34,35] but improved health information systems providing robust research capacity can address this issue [36]. Flexible strategies that promote interoperability of systems may help establish national databases that, if integrated with a vital registration system, may provide insights into long terms outcomes and an effective platform for disease and hospital quality surveillance [37].

### Strengths and Limitations

The strengths of our study include: a novel approach in the creation of a large database with information on three decades, in a resource-limited, low-middle income country, completeness of the dataset and rigorous multiple quality control processes implemented during the data discovery, extraction and preparation phase which allowed for a higher validity and accuracy.

Conversely, there are several important limitations of our study. First, a very detailed variable list could not be created as data systems did not capture characteristics such as symptoms, duration of symptoms, or reasons for not undergoing PCI or other cardiac procedures. However, even with a lean variable list we uncovered several important observations that can then be evaluated comprehensively in a prospective manner. Second, although these data encompass a diverse population cohort, this study is still restricted to a single centre, and may not be generalizable to the entire country. However, it reports on one of the few large-scale electronic health care systems in the country. In-hospital AMI mortality at AKUH may be lower than other health facilities in Pakistan related to variability of capacity in rural and urban set ups. Third, we are unable to make causal associations with the observed temporal trends in mortality. While we can speculate that an increase in revascularization, coupled with adoption of guideline directed medical therapy, has likely played an important role, we cannot comment on adequacy of either therapy. Fourth, we are unable to account for miscoding (if any) of type 2 MI as a primary diagnosis; however, it is likely that this is a small number in the overall cohort.

## Conclusions

AMI presentations and patterns have changed over the last three decades, with a decline in in-hospital mortality in Pakistan. But there are still several care areas such as the use of PCI that need to be further investigated and improved upon. The use of large electronic data to gain insights about disease patterns is feasible in low-middle income countries.

## References

[1] Chandrashekhar Y, Alexander T, Mullasari A, et al. Resource and Infrastructure-Appropriate Management of ST-Segment Elevation Myocardial Infarction in Low- and Middle-Income Countries. Circulation. 2020; 141: 2004–25. DOI: 10.1161/CIRCULATIONAHA.119.041297 32539609

[2] Kristoffersen DT, Helgeland J, Clench-Aas J, Laake P, Veierod MB. Comparing hospital mortality – How to count does matter for patients hospitalized for acute myocardial infarction (AMI), stroke and hip fracture. BMC Health Serv Res. 2012; 12. DOI: 10.1186/1472-6963-12-364 23088745 PMC3526398

[3] Cimci M, Witassek F, Radovanovic D, et al. Temporal trends in cardiovascular risk factors’ prevalence in patients with myocardial infarction. Eur J Clin Invest. 2021; 51: e13466. DOI: 10.1111/eci.13466 33258133

[4] Shah B, Bangalore S, Gianos E, et al. Temporal trends in clinical characteristics of patients without known cardiovascular disease with a first episode of myocardial infarction. Am Heart J. 2014; 167: 480–488.e1. DOI: 10.1016/j.ahj.2013.12.019 24655696 PMC3964370

[5] Seneviratne MG, Kahn MG, Hernandez-Boussard T. Merging heterogeneous clinical data to enable knowledge discovery. Pacific Symp. Biocomput. vol. 24, World Scientific Publishing Co. Pte Ltd. DOI: 10.1142/9789813279827_0040 PMC6447393 30864344

[6] Hertz JT, Reardon JM, Rodrigues CG, et al. Acute myocardial infarction in sub-Saharan Africa: the need for data. PLoS One. 2014; 9: e96688. DOI: 10.1371/journal.pone.0096688 24816222 PMC4016044

[7] Akhlaq A, McKinstry B, Muhammad K Bin, Sheikh A. Barriers and facilitators to health information exchange in low- and middle-income country settings: A systematic review. Health Policy Plan. 2016; 31: 1310–25. DOI: 10.1093/heapol/czw056 27185528

[8] Aluisio AR, Waheed S, Cameron P, et al. Clinical emergency care research in low-income and middle-income countries: Opportunities and challenges. BMJ Glob Heal. 2019; 4. DOI: 10.1136/bmjgh-2018-001289 PMC6666826 31406600

[9] Pakistan Bureau of Statistics. Population Census 2017. http://www.pbs.gov.pk/content/population-census (accessed 2 January 2021).

[10] Rasheed MA, Kedzierski JT, Hasan BS. Improved Family Experience Outcomes in a Pediatric Hospital in Pakistan: Mentoring, Human-Centered Practice, and Theory of Change. NEJM Catal. 2021; 2. DOI: 10.1056/CAT.21.0099

[11] Chan WC, Wright C, Tobias M, Mann S, Jackson R. Explaining trends in coronary heart disease hospitalisations in New Zealand: Trend for admissions and incidence can be in opposite directions. Heart. 2008; 94: 1589–93. DOI: 10.1136/hrt.2008.142588 18519549

[12] Yusuf S, Rangarajan S, Teo K, et al. Cardiovascular risk and events in 17 low-, middle-, and high-income countries. N Engl J Med. 2014; 371: 818–27. DOI: 10.1056/NEJMoa1311890 25162888

[13] Roth GA, Abate D, Abate KH, et al. Global, regional, and national age-sex-specific mortality for 282 causes of death in 195 countries and territories, 1980–2017: a systematic analysis for the Global Burden of Disease Study 2017. Lancet. 2018; 392: 1736–88. DOI: 10.1016/S0140-6736(18)32203-7 30496103 PMC6227606

[14] Ward MJ, Kripalani S, Zhu Y, et al. Incidence of emergency department visits for ST-elevation myocardial infarction in a recent six-year period in the United States. Am J Cardiol. 2015; 115: 167–70. DOI: 10.1016/j.amjcard.2014.10.020 25465931 PMC4276502

[15] Anand S, Bradshaw C, Prabhakaran D. Prevention and management of CVD in LMICs: Why do ethnicity, culture, and context matter? BMC Med. 2020; 18. DOI: 10.1186/s12916-019-1480-9 31973762 PMC6979081

[16] Rogers WJ, Frederick PD, Stoehr E, et al. Trends in presenting characteristics and hospital mortality among patients with ST elevation and non-ST elevation myocardial infarction in the National Registry of Myocardial Infarction from 1990 to 2006. Am Heart J. 2008; 156: 1026–34. DOI: 10.1016/j.ahj.2008.07.030 19032996

[17] Szummer K, Wallentin L, Lindhagen L, et al. Improved outcomes in patients with ST-elevation myocardial infarction during the last 20 years are related to implementation of evidence-based treatments: experiences from the SWEDEHEART registry 1995–2014. Eur Heart J. 2017; 38: 3056–65. DOI: 10.1093/eurheartj/ehx515 29020314 PMC5837507

[18] Wu J, Hall M, Dondo TB, et al. Association between time of hospitalization with acute myocardial infarction and in-hospital mortality. Eur Heart J. 2019; 40: 1214–21. DOI: 10.1093/eurheartj/ehy835 30698766 PMC7618232

[19] Gholap NN, Khunti K, Davies MJ, Bodicoat DH, Squire IB. Survival in South Asian and White European patients after acute myocardial infarction. Heart. 2015; 101: 630–6. DOI: 10.1136/heartjnl-2014-305730 25673527

[20] Mehta A, Singh S, Saeed A, et al. Pathophysiological Mechanisms Underlying Excess Risk for Diabetes and Cardiovascular Disease in South Asians: The Perfect Storm. Curr Diabetes Rev; 2020. DOI: 10.2174/1573399816666200703182458 32619174

[21] Ahmed ST, Rehman H, Akeroyd JM, et al. Premature Coronary Heart Disease in South Asians: Burden and Determinants. Curr Atheroscler Rep. 2018; 20: 6. DOI: 10.1007/s11883-018-0706-1 29374801

[22] Puymirat E, Simon T, Cayla G, et al. Acute myocardial infarction: Changes in patient characteristics, management, and 6-month outcomes over a period of 20 years in the FAST-MI program (French registry of acute ST-elevation or non-ST-elevation myocardial infarction) 1995 to 2015. Circulation. 2017; 136: 1908–19. DOI: 10.1161/CIRCULATIONAHA.117.030798 28844989

[23] Sim DS, Jeong MH. Differences in the Korea Acute Myocardial Infarction Registry Compared with Western Registries. Korean Circ J. 2017; 47: 811. DOI: 10.4070/kcj.2017.0027 29035427 PMC5711672

[24] Zeymer U, Bueno H, Granger CB, et al. Acute Cardiovascular Care Association position statement for the diagnosis and treatment of patients with acute myocardial infarction complicated by cardiogenic shock: A document of the Acute Cardiovascular Care Association of the European Society of Car. Eur Hear Journal Acute Cardiovasc Care. 2020; 9: 183–97. DOI: 10.1177/2048872619894254 32114774

[25] International Diabetes Federation. IDF Diabetes Atlas, 10th edn. Brussels, Belgium; 2021.

[26] Basit A, Fawwad A, Qureshi H, Shera AS. Prevalence of diabetes, pre-diabetes and associated risk factors: second National Diabetes Survey of Pakistan (NDSP), 2016–2017. BMJ Open. 2018; 8: e020961. DOI: 10.1136/bmjopen-2017-020961 PMC6078264 30082350

[27] Cui Y, Hao K, Takahashi J, et al. Age-specific trends in the incidence and in-hospital mortality of acute myocardial infarction over 30 years in Japan — Report from the miyagi ami registry study. Circ J. 2017; 81: 520–8. DOI: 10.1253/circj.CJ-16-0799 28154296

[28] Greco C, Rosato S, D’Errigo P, Mureddu GF, Lacorte E, Seccareccia F. Trends in mortality and heart failure after acute myocardial infarction in Italy from 2001 to 2011. Int J Cardiol. 2015; 184: 115–21. DOI: 10.1016/j.ijcard.2015.01.073 25700282

[29] Yeh RW, Sidney S, Chandra M, Sorel M, Selby JV, Go AS. Population Trends in the Incidence and Outcomes of Acute Myocardial Infarction. N Engl J Med. 2010; 362: 2155–65. DOI: 10.1056/NEJMoa0908610 20558366

[30] Rehman S, Li X, Wang C, Ikram M, Rehman E, Liu M. Quality of care for patients with acute myocardial infarction (AMI) in pakistan: A retrospective study. Int J Environ Res Public Health. 2019; 16. DOI: 10.3390/ijerph16203890 PMC6844119 31615067

[31] Sitthi-Amorn C, Somrongthong R. Strengthening health research capacity in developing countries: a critical element for achieving health equity. BMJ. 2000; 321: 813–7. DOI: 10.1136/bmj.321.7264.813 11009525 PMC1118622

[32] Noorali AAA, Thobani H, Hashmi S, et al. Comparative Trends in Ischemic Heart Disease Admissions, Presentation and Outcomes Due to the COVID-19 Pandemic: First Insights From a Tertiary Medical Center in Pakistan. Cureus. 2021; 13: e17558. DOI: 10.7759/cureus.17558 34646615 PMC8480226

[33] Azubuike MC, Ehiri JE. Health information systems in developing countries: Benefits, problems, and prospects. J R Soc Promot Health. 1999; 119: 180–4. DOI: 10.1177/146642409911900309 10518358

[34] Qureshi NQ, Mufarrih SH, Bloomfield GS, et al. Disparities in Cardiovascular Research Output and Disease Outcomes among High-, Middle- and Low-Income Countries – An Analysis of Global Cardiovascular Publications over the Last Decade (2008–2017). Glob Heart. 2021; 16: 4. DOI: 10.5334/gh.815 33598384 PMC7845477

[35] Hanna TP, Kangolle AC. Cancer control in developing countries: Using health data and health services research to measure and improve access, quality and efficiency. BMC Int Health Hum Rights. 2010; 10. DOI: 10.1186/1472-698X-10-24 20942937 PMC2978125

[36] Bukachi F, Pakenham-Walsh N. Information technology for health in developing countries. Chest. 2007; 132: 1624–30. DOI: 10.1378/chest.07-1760 17998362

[37] Braa J, Hanseth O, Heywood A, Mohammed W, Shaw V. Developing Health Information Systems in Developing Countries: The Flexible Standards Strategy. MIS Q. 2007; 31: 381–402. DOI: 10.2307/25148796

